# Intracranial CNS Manifestations of Myeloid Sarcoma in Patients with Acute Myeloid Leukemia: Review of the Literature and Three Case Reports from the Author’s Institution

**DOI:** 10.3390/jcm4051102

**Published:** 2015-05-21

**Authors:** Gustavo M. Cervantes, Zuzan Cayci

**Affiliations:** Department of Neuroradiology, University of Minnesota, Minneapolis, MN 55455, USA; E-Mails: cerva039@umn.edu (G.M.C.); cayci001@umn.edu (Z.C.); Tel.: +1-612-626-5566; Fax: +1-612-626-5505

**Keywords:** AML, acute myeloid leukemia, leukemia, myeloid sarcoma, extramedullary disease, chloroma, intracranial, central nervous system, brain, spine

## Abstract

Myeloid sarcoma (MS) of the central nervous system (CNS) is a rare presentation of leukemic mass infiltration outside of the bone marrow. It may involve the subperiosteum and dura mater and, on rare occasions, can also invade the brain parenchyma. The disease is most commonly seen in children or young adults; however, it has been described in multiple age groups. MS can be seen in patients with acute myeloid leukemia (AML), chronic myeloid leukemia and other myeloproliferative disorders. This entity has the potential to be underdiagnosed if the MS appearance precedes the first diagnosis of leukemia. The main reason is that their appearance on CT and MRI has a broad differential diagnosis, and proper diagnosis of MS can only be made if the imaging findings are correlated with the clinical history and laboratory findings. Herein, we describe the intracranial CNS manifestations of MS in patients with AML on CT and MRI involving the brain and/or meninges. This study is based on a systematic review of the literature. In addition, three case reports from the author’s institution with AML and intracranial involvement of MS are included. Our aim is to enhance the awareness of this entity among both clinicians and radiologists.

## 1. Introduction

Central nervous system (CNS) myeloid sarcoma (MS), a rare manifestation of acute myeloid leukemia (AML), chronic myeloid leukemia and other myeloproliferative disorders were first described by Burns in 1811 [1] by the term chloroma. MS can develop when the immature myeloblast groups form solid tumors outside the bone marrow. Intracranially, myeloid sarcomas are often continuous with the meninges or the ependyma. Nevertheless, on rare occasions, myeloid sarcoma may invade the brain parenchyma and, thus, may appear as intra-axial masses.

According to Audouin *et al.*, [2], there are four different patterns of myeloid sarcoma development in AML patients:

(1) They may develop during the active phase of leukemia;
(2) They may develop concurrently with known chronic myeloproliferative disorders;
(3) They may manifest as a relapse after months or years after clinical remission of AML, particularly after bone marrow transplantation;
(4) They may precede the AML diagnosis and may be detected in previously healthy patients who have a normal peripheral blood cell count and who have no blast infiltration in the bone marrow (0.6% of cases as described by Krause [3]). In this group of patients, most of the patients develop myeloid leukemia blast infiltration an average of 10.5 months following the diagnosis of a myeloid sarcoma [4]. In a large population-based cohort study from Denmark, the prevalence of myeloid sarcoma among 2261 patients with AML was 9.7%; however, CNS involvement by myeloid sarcoma was only present in 0.4% of these patients [5].

In this review of the literature, we have identified 21 reported cases of myeloid sarcoma in patients with AML and describe the diagnostic and imaging findings pertinent to the brain parenchyma and/or meninges. In addition, three patients from our institution with AML and positive intracranial involvement were included.

## 2. Literature Review

A PubMed database search using the descriptors chloroma, myeloid sarcoma, myeloid sarcoma and brain, intracranial, central nervous system and cranial nerves retrieved 56 relevant citations from 1971 to 2014. Reviewing the literature, 21 reported cases of myeloid sarcoma with a sufficient description of the diagnosis and imaging findings related to the brain and/or meninges were identified. Leukemic conditions other than AML, such as chronic myeloid leukemia and myeloproliferative disorders, were not included. Only articles in English that were available on the PubMed central publishing platform were considered.

## 3. Results

CT and MRI were the most commonly utilized imaging modalities for assessment of CNS myeloid sarcoma. Solely MR imaging was used in 11 reported cases, and solely CT was used in four case reports. Combined MR and CT were utilized in six reported cases. Intracranial CNS imaging manifestations of myeloid sarcomas were reviewed in regards to their anatomic site, intra-axial *versus* extra-axial location, single *versus* multiple lesions and CT and/or MR imaging characteristics (Table 1).

A total of 21 reported patients (ten male and ten female) with AML and CNS myeloid sarcoma were included in this review. Gender was not available for one case report [6]. Nineteen patients included were adults, one a three-year-old child and another a 16-year-old adolescent. Patients’ ages ranged between three and 58 years old. Mean age at the time of diagnosis of intracranial myeloid sarcoma was 35 years. Out of the 21 reported cases, 13 patients (61%) had preexisting AML, either in remission or in acute bone marrow relapse at the time of the first neurologic symptoms. Eight patients (38%) presented with CNS symptoms preceding their AML diagnosis.

Out of the 21 patients, a total of 24 intracranial myeloid sarcoma lesions were described. Of those, 13 lesions (54%) were described in the intra-axial compartment of the brain and 11 lesions (45%) in the extra-axial brain compartment. One patient had sequential lesions develop in more than one anatomic site in the brain [7]. Another patient had concurrent intra-axial lesions described in the left occipital lobe, right temporal lobe and left cerebellum [8].

Overall, the sites of intracranial myeloid sarcomas included the temporal lobe (*n* = 6), frontal lobe (*n* = 4), cerebellum (*n* = 4), parietal lobe (*n* = 3), occipital lobe (*n* = 3), cerebellopontine angle cistern (*n* = 1), corpus callosum (*n* = 1), basal ganglia (*n* = 1) and subdural space (*n* = 1). Six patients described also showed either concurrent or sequential lesions in locations outside the brain. These extra-cranial locations included the temporal bone (*n* = 1), both kidneys (*n* = 1), multiple bones (*n* = 1), nasopharynx (*n* = 1), cervical and lumbar spine (*n* = 1), thoracic and sacral spine (*n* = 1), scalp tissues (*n* = 2) and infratemporal fossa (*n* = 2). One intracranial vascular lesion involving the superior sagittal sinus was described in one patient (*n* = 1).

Twelve out of a total of 24 lesions were assessed with CT. Among these, 11 lesions (91%) appeared hyperdense on noncontrast CT. Out of all 11 hyperdense lesions, six lesions were intra-axial and five extra-axial. Twenty-two lesions (91%) exhibited avid homogeneous enhancement. One lesion demonstrated inhomogeneous thick peripheral enhancement, indistinguishable from a brain abscess [9]. In another patient, a lesion described in the right temporal lobe demonstrated avid enhancement with a central core of hypoenhancement, suggestive of necrosis [8].

Seven intra-axial MS lesions were assessed with brain MRI. MS demonstrated either a hyper, iso- or hypo-intense signal on T2-weighted images. T2 hyperintensity was described in four lesions, while T2 iso- or hypo-intensity was described in three lesions. Nine extra-axial MS lesions were assessed with MRI. T2 hyperintensity was described in two extra-axial lesions; however, T2 signal findings were not described for the remaining seven extra-axial reported lesions assessed with MRI. Primary bone destruction was evident in one patient, resulting in erosion of the petrous portion of the temporal bone and subsequent intraparenchymal involvement of the right temporal lobe [10].

Vasogenic edema surrounding the enhancing leukemic masses was identifiable on both CT and MRI. This was seen in 11 lesions (45%). Among these, six lesions were in the extra-axial and five in the intra-axial compartment of the brain.

**Table 1:** Summary of clinical findings, CT and MR features of intracranial myeloid sarcomas (MS) in 21 reported cases presenting with acute myeloid leukemia.

Authors, Year	Age (Years), Gender	AML Diagnosis	Single *vs.* Multiple	CNS Anatomic Location (s)	Intra- or Extra-Axial	CT and/or MR Characteristics
Yang *et al.*, 2014 [11]	27, male	preceding AML diagnosis	single	occipital lobe (1), spine (2)	extra-axial	meningioma-like, vasogenic edema
Cho *et al.*, 2013 [12]	27, female	AML relapse, 10 months in CR	single	right cerebellopontine angle	extra-axial	meningioma-like, vasogenic edema
Murakami *et al.*, 2011 [10]	52, male	AML relapse, 5 years in CR	multiple	right temporal bone and right temporal lobe	extra-axial	meningioma-like, vasogenic edema
Akhaddar *et al.*, 2011 [6]	27, n/a	preceding AML diagnosis	single	right frontal lobe	intra-axial	intraparenchymal, vasogenic edema
Eom *et al.*, 2011 [7]	49, male	AML relapse, 2 years in CR	multiple	left cerebellum, right frontal lobe and cervical and lumbar spine	intra-axial	intraparenchymal
Cho *et al.*, 2010 [13]	44, male	AML relapse, 2 years in CR	single	corpus callosum	intra-axial	meningioma-like
Grier *et al.*, 2008 [14]	41, female	preceding AML diagnosis	multiple	left temporal lobe, infratemporal extension	extra-axial	meningioma-like
Widhalm *et al.*, 2006 [15]	35, female	preceding AML diagnosis	multiple	right parietal lobe and subgaleal (1), spine (2)	extra-axial	meningioma-like
Smidt *et al.*, 2005 [16]	45, male	AML relapse, 16 years in CR	single	left cerebral hemisphere subdural location	extra-axial	hemispheric subdural mass, C+, mass effect
Best-Aguilera *et al.*, 2005 [17]	37, male	preceding AML diagnosis	multiple	right occipital lobe, mediastinum, retroperitoneum, liver, rectum	extra-axial	meningioma-like, vasogenic edema
Nishimura *et al.*, 2004 [18]	30, female	AML relapse, 16 months in CR	single	right frontal lobe, subgaleal and superior sag. sinus extension	extra-axial	meningioma-like, vasogenic edema
Suzer *et al.*, 2004 [19]	58, female	AML relapse, 6–12 months in CR	single	left cerebellar hemisphere	intra-axial	Intraparenchymal
Park *et al.*, 2003 [20]	3, female	preceding AML diagnosis	multiple	right temporal lobe, infratemporal extension, kidneys, bones	extra-axial	meningioma-like
Nikolic *et al.*, 2003 [21]	45, male	AML relapse, indeterminate CR	single	left frontal lobe	extra-axial	meningioma-like, vasogenic edema
Guermazi *et al.*, 2002 [22]	28, female	AML relapse, 8 months in CR	single	left parietal lobe	intra-axial	intraparenchymal, vasogenic edema
Guermazi *et al.*, 2002 [22]	58, female	preceding AML diagnosis	single	right basal ganglia	intra-axial	intraparenchymal, vasogenic edema
Ooi *et al.*, 2001 [9]	32, male	AML relapse, 18 months in CR	multiple	right temporal lobe, nasopharynx	intra-axial	intraparenchymal, mimicking abscess
Yamamoto *et al.*, 1999 [23]	38, male	AML relapse, 2 years in CR	single	left temporal lobe	intra-axial	intraparenchymal, vasogenic edema
Parker *et al.*, 2005 [24]	29, female	AML relapse, 6 months in CR	single	cerebellar vermis	intra-axial	intraparenchymal
Yoon *et al.*, 1987 [25]	16, female	preceding AML diagnosis	single	left parietal lobe	extra-axial	meningioma-like
Barnett, Zussman 1986 [8] *	34, male	AML relapse, 13 months in CR	multiple	left occipital and left cerebellum	intra-axial (2)	intraparenchymal lesions
Barnett, Zussman 1986 [8] *	34, male	AML relapse, 13 months in CR	multiple	right temporal lobe	intra-axial (1)	intraparenchymal, hypodense lesion, core of necrosis

Asterisks (*) represent a case report from the literature with three concurrent lesions described, which were separated into two paragraphs, because of the peculiar imaging findings of the right temporal lobe lesion; numbers in parentheses represent numerical values of the lesions described; n/a, not available.

## 4. Authors’ Institution Case Reports

CT and MR imaging characteristics of three patients from our institution presenting with acute myeloid leukemia and intracranial CNS manifestations of myeloid sarcoma are described. Case 3 was previously reported in the literature by one of the authors [26].

**Figure 1:**
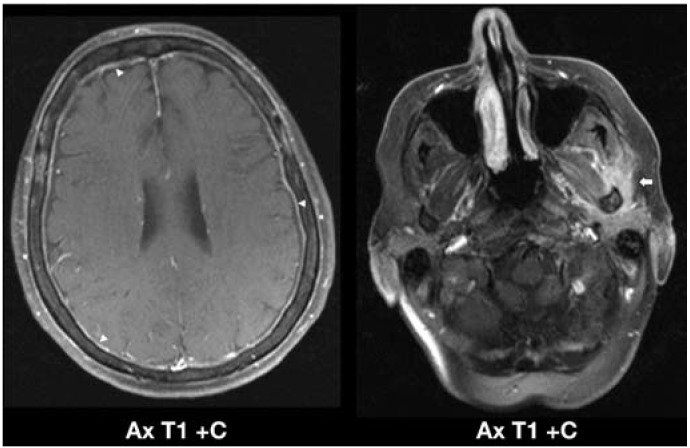
Meningeal infiltration related to AML in a previously healthy 69-year-old woman. Axial post-contrast T1-weighted images with fat saturation (**left image**) demonstrate marked pachymeningeal enhancement (arrowheads), most consistent with leukemic infiltration of the brain meninges. Note the marked ill-defined enhancement of the left infratemporal fossa (**right image**) surrounding the left masticator and deep parotid spaces (arrow), corresponding to extra-cranial leukemic myeloid sarcoma of the soft tissues of the left neck. Complete resolution of the leukemic infiltration within the deep left neck was observed after successful AML induction chemotherapy on follow-up imaging (not shown). Ax, axial; C+, post-contrast.

Case 1: A 69-year-old female patient with a recent history of worsening headaches and flu-like symptoms diagnosed with AML and more than 90% myeloid cells in the blood count during her first admission (Figure 1).

**Figure 2:**
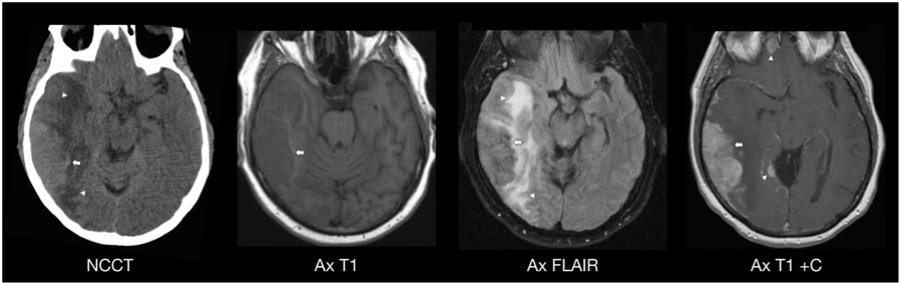
AML relapse in the form of a myeloid sarcoma mass lesion involving the right temporal lobe of a 56-year-old woman 39 days after umbilical cord blood stem cell transplantation. Noncontrast CT images demonstrate a large hyperdense dural-based mass lesion involving the right temporal lobe (NCCT, arrow) with surrounding edema (NCCT, arrowheads), mass effect and midline shift. On MRI, a large ill-defined avidly enhancing dural-based mass lesion involving the posterior aspect of the right temporal lobe (Ax T1 C+, arrow) with surrounding vasogenic edema (Ax FLAIR, arrowheads) was noted. This infiltrating mass revealed hypointense signal intensity on T1 (Ax T1, arrow) and T2-weighted images relative to the adjacent gray-matter. Diffusion weighted images (not shown) demonstrated restricted diffusion in the posterolateral aspect of the right temporal lobe mass consistent with increased cellularity. Additional pachymeningeal foci of nodular enhancement were noted within the gyrus rectus of the right inferior frontal lobe and within the medial aspect of the right inferior temporal lobe (Ax T1 C+, arrowheads). Diffuse right hemispheric pachymeningeal enhancement was also noted (Ax T1 C+, partially shown). Ax, axial; Sag, sagittal; FS, fat saturation; C+, post-contrast; DWI, diffusion-weighted images; ADC, apparent diffusion coefficient.

Case 2: A 56-year-old female patient with acute myelomonocytic leukemia, French-American-British (FAB) classification M4, developed a first relapse of AML with 26% blasts in the blood count during admission. She underwent double umbilical cord blood stem cell transplantation during the remission phase after being successfully treated with induction chemotherapy for her first relapse. On Day 39 of the post-transplant period, she presented with persistent headaches, muscle weakness, falls at home and the onset of leukemia cutis with new skin lesions (Figure 2).

**Figure 3:**
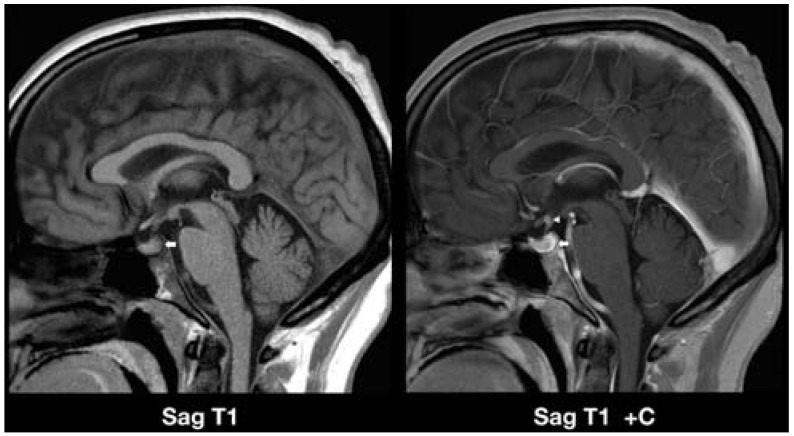
Leukemic infiltration of the neurohypophysis and pituitary stalk in a patient with myelodysplastic syndrome presenting with diabetes insipidus. The sagittal T1-weighted image without contrast administration (**left image**) demonstrates the absence of the neurohypophysis (arrow), confirming the diagnosis of diabetes insipidus. Normally, the neurohypophysis is identified as a bright signal spot on T1-weighted images in the posterior pituitary gland. The sagittal T1-weighted image after contrast administration **(right image**) reveals a 2-mm enhancing nodular lesion in the superior aspect of the pituitary stalk (arrowhead) and curvilinear enhancement along the posterior aspect of the pituitary gland in the expected region of the neurohypophysis (arrow). Sag, sagittal; C+, post-contrast.

Case 3: A 53-year-old man with a three-month history of polydipsia and polyuria and indication of myelodysplastic syndrome (MDS) presented with refractory anemia and 16% blast cells in the bone marrow biopsy suggesting a diagnosis of refractory anemia with excess of blasts (RAEB). Diabetes insipidus was confirmed by inappropriately low urine osmolality and low antidiuretic hormone (ADH) levels. AML induction chemotherapy (seven days of cytarabine and three days of idarubicin) along with intrathecal methotrexate after cerebrospinal fluid (CSF) analysis was initiated (Figure 3).

## 5. Discussion

CNS leukemic infiltration may present in one or more of the following intracranial forms: (1) meningeal disease, as “carcinomatous meningitis”; (2) intravascular tumor aggregates throughout the brain, as “carcinomatous encephalitis”; or (3) focal tumor masses, as “myeloid sarcomas”. When AML manifests as a solid tumor outside the bone marrow, *i.e.*, myeloid sarcoma, the imaging features can be mistaken for some conditions other than the myeloid sarcoma. Myeloid sarcoma seen in leukemic patients may involve any part of the body. The most common sites for myeloid sarcoma deposits are the bones, soft tissues, lymph nodes and the skin. Rarely, they may manifest as single or multiple intracranial lesions [4], most commonly seen within the calvaria and orbits [27]. In our review, out of 24 intracranial MS lesions reviewed from the literature, 13 lesions (48%) have presented as focal tumor masses within the intraparenchymal compartment of the brain.

Myeloid sarcoma can occur at variable ages (1–81 years old), often after the diagnosis of AML. However, in approximately 25% of the patients, MS appears before the initial diagnosis of AML by months or years [28]. According to Neiman *et al.*, of a total of 61 biopsied proven myeloid sarcomas, twenty-two lesions were seen in 15 patients with no known history of acute myeloid leukemia [4]. Most of these patients developed acute leukemia on an average of 10.5 months after the biopsy. In this review, eight out of 21 reported cases (38%) had intracranial CNS imaging manifestations of myeloid sarcoma preceding the diagnosis of AML.

Radiologically, intracranial MS in patients with acute myeloid leukemia most commonly presents as an extra-axial hyperdense mass on noncontrast CT scan. In this review of the literature, all 24 intracranial lesions, except for one, appear as hyperdense masses on the noncontrast CT studies. The differential diagnosis of hyperdense intracranial masses is broad and includes meningioma, B-cell lymphoma and intracranial metastasis. Interestingly, myeloid sarcomas can express B-cell antigens (e.g., CD19, CD79a) and, thus, potentially lead to a histologic misdiagnosis of CNS lymphoma, if no immunohistochemistry or flow cytometry analysis is available [14]. Other less common diagnostic considerations considered are metastatic neuroblastoma (in the pediatric age group), Ewing’s sarcoma, hemangiopericytoma and subdural hematoma. A second pattern of presentation is patchy and/or leptomeningeal enhancement seen in patients with acute myeloid leukemia. In this situation, the differential diagnosis to consider is: primary meningeal tumor infiltration by leukemia, secondary leptomeningeal carcinomatosis, viral or bacterial meningitis, neurosarcoidosis, dural sinus thrombosis, intracranial hypotension or postoperative changes.

Vasogenic edema was present on either CT or MR in 11 lesions (45%) out of the 24 intracranial myeloid sarcomas that were included in this review. Further characterization of other CT and/or MR imaging features should be used to distinguish the intracranial MS lesions from other commonly-found intracranial lesions, such as meningioma, lymphoma or brain metastasis, which also commonly show vasogenic edema and can potentially occur concurrently with myeloid sarcoma in patients with acute myeloid leukemia.

Leukemic cell infiltrates are capable of migration from the bone marrow of the periosteum and dura mater into the underlying brain parenchyma once there is disruption of the pial-glial barrier. Despite the close involvement of the extra-cranial and intra-cranial tissues, destructive bony changes are not commonly seen with myeloid sarcomas. Only one patient showed apparent bone destruction of the temporal bone and concomitant involvement of the adjacent right temporal lobe parenchyma [10].

One of the limitations of this study is the small sample of the reported cases available in the literature, since we exclusively included AML patients with intracranial MS lesions. Other forms of acute or chronic leukemias and myeloproliferative disorders could also present with intracranial MS lesions and were not included in this study. Potential selection bias of the study population is another limitation, since the majority of patients included were young adults. Since only one pediatric patient with AML and intracranial CNS myeloid sarcoma was present in our systematic review, our findings cannot be generalized to all age groups.

## 6. Conclusions

Myeloid sarcoma of the intracranial CNS is a rare presentation of AML. This can be detected before the diagnosis of leukemia or any time throughout the course of the disease during routine neuroimaging studies. Proper CT and MR imaging interpretation in conjunction with relevant clinical information, including the age at the time of diagnosis, symptomatology, such as the presence of headaches, seizures, focal neurological deficits and/or cranial nerve palsy (-ies), a history of hematologic malignancies, prior bone marrow or solid organ transplantation status and immune system status, can help to narrow the clinical diagnosis of intracranial MS. Finally, prompt clinical and laboratory evaluation for acute myeloid leukemia relapse is necessary in patients with a known history of leukemia presenting with meningeal and/or parenchymal lesions.
